# Poison Gases in Trench Warfare

**Published:** 1917-09

**Authors:** 


### POISON GASES IN TRENCH WARFARE.

The exact composition of the gases used in modern trench warfare is not known, but from the appearance, odor, and effects on the men it would seem that most commonly a mixture of chlorine and bromine is employed; possibly at times with the addition of sulphur fumes or formaldehyde gas. Chlorine and bromine are produced cheaply and in large amounts by the Germans as by-products of other industries. They are among the most active chemical agents known, attacking the eyes, and lining of the mouth, throat and nose. They first produce a hard cough, followed by the spitting of blood, and, finally asphyxiation, due to the destruction of the breathing apparatus. Only one part of chlorine or bromine in one thousand parts of air is necessary to produce almost instant death; one part in one hundred thousand, if endured for any great length of time, is very dangerous.

For use in the trenches the gases are usually liquified and stored in tanks from which the outflow is regulated by means of a valve. If the ground slopes a little toward the enemy and the wind is in the right direction, the gas, being heavier than air, flows over the ground, filling the hollows like so much water.

The most successful method for combating the gas attacks is by the use of a gas mask. The modification now employed is a hood provided with a mica window that fits down over the head like a bag, buttoning between the vest and shirt. When the first indications of an attack are evident the hood is moistened with a solution of sodium hyposulphite (the photographer's ''hypo''), which combines with the gases, rendering them ineffective. Because of the large amount of gas required to poison the constantly changing air an attack is only of a few minutes duration. In case one is overcome by the gas, inhalation of dilute ammonia vapors will give great relief, since the ammonia combines with the gas in the bronchial tubes and relieves the difficulty of breathing, although it does not undo the injury already done.

Because of the cruel suffering inflicted upon the enemy the use of poisonous gases in projectiles was foresworn by the signers of the Hague declaration in 1899. The first gas attacks of the Germans took the enemy by surprise and inflicted great losses and an untold amount of suffering. Since then the masks have been so perfected that the troops have lost most of their fear of this ruthless form of battle, thus again emphasizing the fact that this is a war fought by science.—Pharmaceutical Era.

